# Dual endovascular repair (coils and stent) of a true aneurysm of the gastroduodenal artery

**DOI:** 10.1590/1677-5449.190123

**Published:** 2020-08-31

**Authors:** Carola Rubio Taboada, Jesús García Alonso, Rubén Peña Cortés, Luis Velasco Pelayo, Paula Velasco Hernández, Francisco Santiago Lozano Sánchez

**Affiliations:** 1 Hospital Clínico de Salamanca, Angiología y Cirugía Vascular, Salamanca, España.; 2 Hospital Clínico de Salamanca, Radiología, Salamanca, España.

**Keywords:** endovascular treatment, aneurysm, gastroduodenal artery, tratamento endovascular, aneurisma, artéria gastroduodenal

## Abstract

We report a case of an asymptomatic gastroduodenal artery aneurysm diagnosed in a 39 year-old woman. An abdominal ultrasound study showed an aneurysmal dilatation of the gastroduodenal artery with 2 x 2 cm diameter. To confirm this finding, she then underwent a computed tomography scan of the abdomen and pelvis that showed a saccular aneurysm of the gastroduodenal artery. A dual endovascular approach was used to exclude the aneurysm by stent-assisted coil embolization. Complete exclusion of the aneurysm sac was confirmed on final angiography. She was discharged from the hospital on postoperative day 1.

## Resumo

Relatamos um caso de aneurisma de artéria gastroduodenal assintomático diagnosticado em uma mulher de 39 anos. Uma ultrassonografia abdominal mostrou uma dilatação aneurismática associada à artéria gastroduodenal com 2 x 2 cm de diâmetro. Para confirmar esse achado, foi realizada uma tomografia computadorizada do abdome e da pelve, que revelou um aneurisma sacular da artéria gastroduodenal. Uma abordagem endovascular dupla foi utilizada para exclusão do aneurisma mediante embolização com molas assistida por stent. A exclusão completa do saco aneurismático foi confirmada na angiografia final. A paciente recebeu alta hospitalar no primeiro dia de pós-operatório.

## INTRODUCTION

Visceral artery aneurysms (VAA) are rare manifestations with incidence between 0.01% and 0.2%.^1^ Splenic artery aneurysms are the most common type, accounting for 60%. These are followed by aneurysms of the hepatic artery (20%); superior mesenteric artery (6%); celiac artery (4%); gastric and gastroepiploic arteries (4%); jejunal, ileal, and colic arteries (3%); pancreaticoduodenal and pancreatic arteries (2%); gastroduodenal artery (GDA) (1.5%); and inferior mesenteric artery, at <1%.^2^^,^^3^ Despite their low incidence, they should be considered important because they have a disproportionate rupture rate (25%) and significant morbidity and mortality (70%).^4^^-^6 Unlike most VAAs, GDA aneurysms tend to be symptomatic. The most common symptom is vague epigastric pain that radiates to the back. Gastroduodenal artery aneurysms (GDAA) are divided into two groups, true aneurysms and pseudoaneurysms (60%). Pseudo-GDAA, secondary to other causes such as pancreatitis, gastric or pancreatic surgery, are seen more frequently and have a higher chance of diagnosis. True aneurysms are much rarer and more difficult to diagnose, so they are more likely to rupture.^7^^-^9 Up to 60% of pancreaticoduodenal aneurysms and GDAA present with rupture;^10^ and should therefore be repaired promptly irrespective of size.^11^ Most of them have historically been managed with open surgery. With the current adoption of endovascular therapies, these aneurysms can now be treated by embolization with or without stent implant, with success rates between 78% and 97%. Open surgery is now reserved for unstable patients and/or ruptured aneurysms and/or failure of endovascular treatment.^12^

The aim of this article is to present a case of a true asymptomatic GDAA that was resolved by endovascular exclusion with stent-assisted coil embolization.

## CASE REPORT

**Figure 1:**
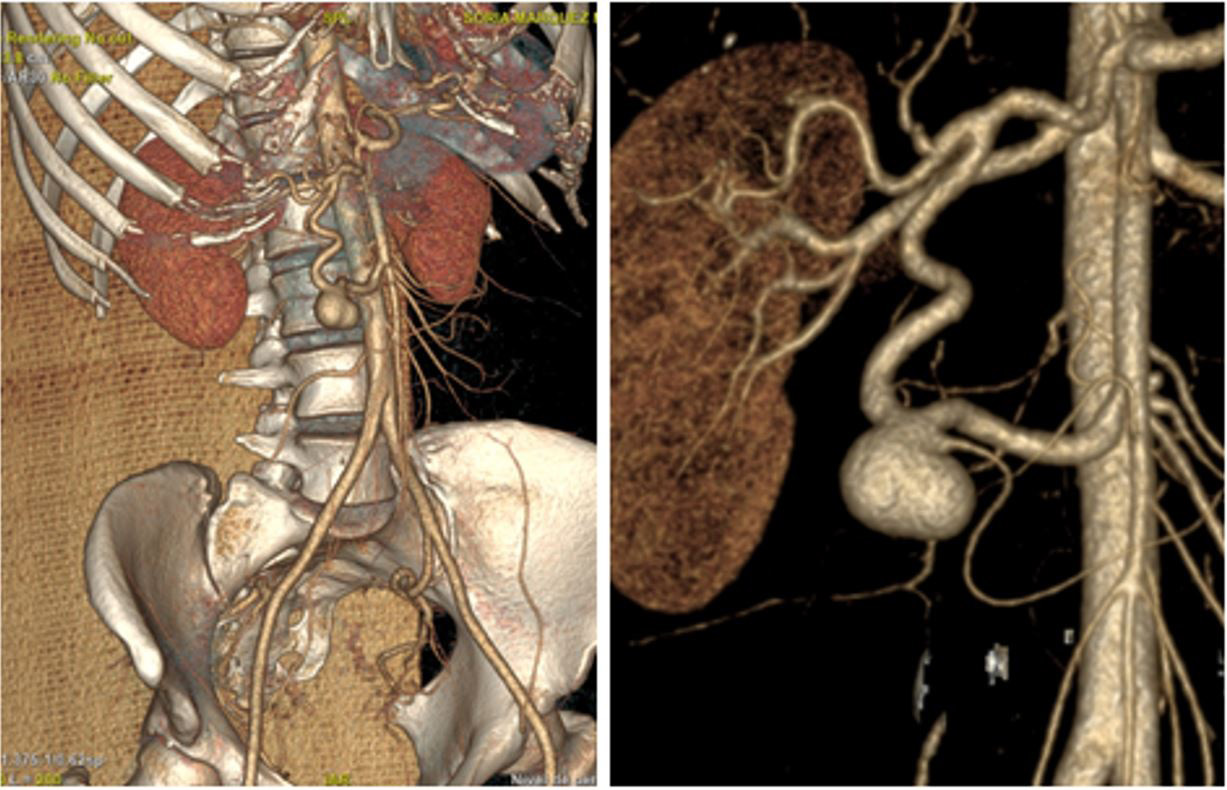
Computed tomography (CT) scan of the abdomen.

**Figure 2:**
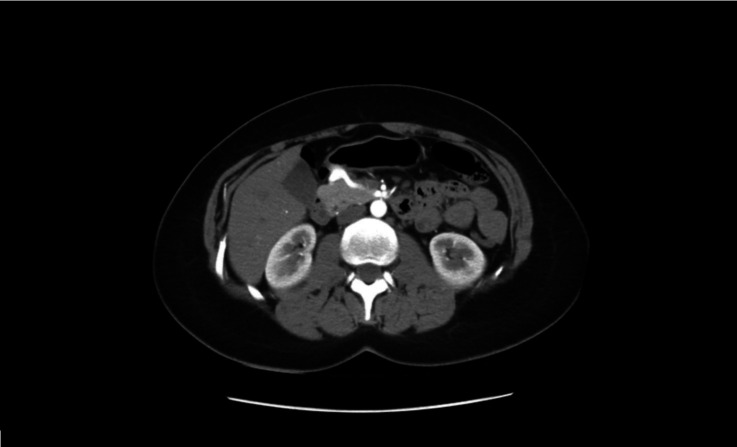
Angiogram shows no flow in the aneurysm sac.

**Figure 3:**
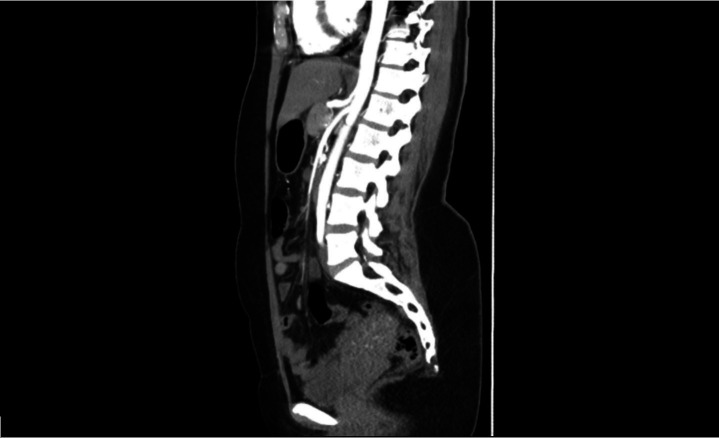
No significant stenosis of the superior mesenteric artery or celiac trunk.

**Figure 4:**
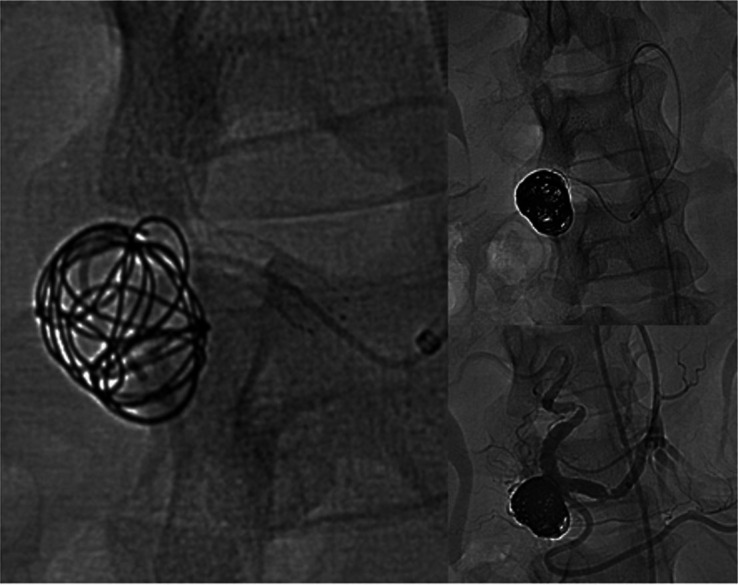
Angiogram shows no flow in the aneurysm sac.

We present the case of a 39-year-old woman with medical history of anxiety and two cesarean deliveries, no history of trauma or any digestive disease including pancreatic pathology, and who did not meet collagen disease criteria. She had a Family history of father in follow-up for an abdominal aortic aneurysm (AAA). Because of her family history and after reporting feeling blood pumping in the epigastric region during an anxiety crisis, an abdominal ultrasound study was performed, which detected an aneurysmal dilatation of the GD artery with 2 × 2 cm diameter and no signs of complications. To confirm this, she then underwent a computed tomography (CT) scan of the abdomen (Figures 1 and 2) that showed a saccular aneurysm of the GD artery with a diameter of 19.7 × 14 × 20 mm and communication with the superior mesenteric artery and no signs of complications. No significant stenosis of the superior mesenteric artery or celiac trunk was found (Figure 3). On the basis of these findings, she was scheduled for endovascular intervention. The aneurysm was excluded by stent-assisted coil embolization using a 5 French sheath. An angiogram was performed, confirming the diagnosis seen on the CT scan and ultrasound study. The patient was systematically heparinized with 4000 IU intravenous (IV) heparin. The GDA was selectively catheterized using a Cobra catheter. Since the aneurysm was saccular with a large neck, we decided to implant an open-cell stent (Biotronik AG® Pulsar-18, Bülach, Switzerland. 6/40/135) and through its cells we deployed multiple detachable coils (Helixev3® Concerto, Irvine, CA, USA. 20 mm × 50 cm: 1; 18 mm × 40 cm: 1; 15 mm × 40 cm: 3; 14 mm × 30 cm: 2; 12 mm × 30 cm: 2; and 10 mm × 30 cm: 1) into the aneurysm sac. A completion angiogram showed no flow in the aneurysm sac (Figure 4). The patient had an uneventful hospital postoperative course and was discharged from hospital on postoperative day 1.

## DISCUSSION

True GDAA are very rare and constitute 1.5% of all VAA.^2^ The first report was published by Starlinger in 1930 and since then there have been increasing numbers of reports of GDAA, due to improved radiological techniques.^5^^,^^9^ They are usually seen between the ages of 50 to 58. The female/male ratio is 1/4.5 and the mean diameter is 3.6 cm.^6^^,^^9^ Although their pathophysiology is not fully understood, GDAA are divided into two groups: true aneurysms and pseudoaneurysms, based on the mechanism of formation: true aneurysms involve all layers of the vessel wall, whereas pseudoaneurysms are false aneurysms that result from injury to one or more vessel wall layers. Hypertension, atherosclerosis and autoimmune diseases such as systemic lupus, Wegener's granulomatosis, and polyarteritis nodosa are possible etiologic factors of true GDAA.^9^ Pseudoaneurysms occur after injury or erosion of vessels due to causes such as trauma and inflammation. The most common cause of pseudoaneurysms is destruction of the vascular wall by proteolytic enzymes released from pancreatitis.^7^^,^^9^ Pancreaticoduodenal and gastroduodenal arteries are the major collateral pathways between the celiac and superior mesenteric arteries. It has been suggested that stenosis or occlusion of celiac and superior mesenteric arteries could cause increased blood flow to the collateral vessels, followed by aneurysms.^5^^,^^9^^,^^13^ In our case, it was observed that both trunks were open.

The most common clinical presentation (52%) is gastrointestinal hemorrhage due to rupture.^14^^,^^15^ The second most common presentation is abdominal pain (46%), while 7.5% of GDAA remain clinically silent.^9^ Patients can also present with symptoms of gastric obstruction, compressive symptoms (nausea, vomiting), hemobilia, and pulsatile abdominal mass. Our patient presented with a feeling of blood pumping in the epigastric region during an anxiety crisis that was not confirm at physical examination.

Most VAA are asymptomatic lesions that are incidentally diagnosed during examinations ordered for an unrelated abdominal pathology.^5^^,^^16^

In symptomatic patients, the diagnostic procedures primarily preferred are ultrasonography and CT because they are non-invasive, and have sensitivity of 50% and 67%, respectively.^9^ The gold standard for diagnosis is catheter angiography. The sensitivity of this procedure is 100%. The most important advantage is that it can be used for treatment.^7^^,^^9^

Once they are diagnosed, even asymptomatic GDAA should be treated, regardless of their size, because of the high morbidity and mortality on account of the potential for rupture (40% mortality rate secondary to rupture).^4^^,^^17^ Most of these lesions have historically been managed with open ligation or aneurysmectomy. With the current adoption of endovascular therapies, these aneurysms can now be treated by embolization with or without stent implant.

Endovascular exclusion is the first-line therapy for stable patients with GDAA and this offers significant advantages in terms of less postoperative pain, shorter hospital stay, and earlier return to activities of daily life.^5^ Surgical treatment (open, laparoscopic,^18^ or robotic) is recommended in cases of hemodynamically unstable patient, failed endovascular repair, or unsuitable anatomy.^19^^,^^20^ However, hemodynamically unstable patients can be treated successfully with endovascular interventions. Aneurysm ruptures, especially those involving bifurcations of the GDA, are located deep in the pancreas and are difficult to detect during surgery. In such cases, early postoperative diagnosis and treatment with endovascular techniques are life-saving.

According to our review, our patient is the first reported case in which an aneurysm of the gastroduodenal artery was treated with a dual endovascular technique (coils and stent). In this case, the endovascular repair was accomplished with no complications and total exclusion of the GDA was confirmed in the final angiography.

## CONCLUSION

True GDAA are very rare and can be detected by chance, as incidental findings on a CT scan or abdominal ultrasound study. The decision to operate should be taken without any delay because of their potential to rupture. Endovascular exclusion is the first-line therapy for stable elective GDAA patients. For ruptured GDAA or unstable patients, the treatment modality will depend on the expertise of the operator, the patient’s comorbidities, and the hospital’s capacity.
